# JMJD8 Functions as a Novel AKT1 Lysine Demethylase

**DOI:** 10.3390/ijms24010460

**Published:** 2022-12-27

**Authors:** Yujuan Wang, Yaoyao Zhang, Zehua Li, Junfeng Wang

**Affiliations:** 1 High Magnetic Field Laboratory, CAS Key Laboratory of High Magnetic Field and Ion Beam Physical Biology, Hefei Institutes of Physical Science, Chinese Academy of Sciences, Hefei 230031, China; 2 Science Island Branch of Graduate School, University of Science and Technology of China, Hefei 230026, China; 3 Institutes of Physical Science and Information Technology, Anhui University, Hefei 230601, China

**Keywords:** JMJD8, demethylase, AKT1, tri-methylated lysine, JmjC domain

## Abstract

JMJD8 is a protein from the JMJD family that only has the JmjC domain. Studies on the function of JMJD8 indicate that JMJD8 is involved in signaling pathways, including AKT/NF-κB, and thus affects cell proliferation and development. Here, we reported the activity of JMJD8 as a non-histone demethylase. We investigated the demethylation of JMJD8 on trimethylated lysine of AKT1 in vivo and in vitro using trimethylated AKT1 short peptide and AKT1 protein, and we tracked the regulation of JMJD8 on AKT1 activity at the cellular level. The results showed that JMJD8, a mini lysine demethylase, altered AKT1 protein function via changing its degree of methylation.

## 1. Introduction

Lysine demethylase (KDM) regulates the methylation level of lysine in specific proteins for post-translational modifications. Most KDMs reported are histone demethylases that contribute significantly to gene expression, chromatin organization and genomic integrity [1,2]. It has been demonstrated that JMJD2 (KDM4) and JMJD3 (KDM6), members of the JMJD protein family with a Jumonji C domain, have histone lysine or arginine demethylase activity (KDM, RDM) [1,2,3,4]. JMJD2, comprising JMJD2A-D proteins, is one of the largest JMJD subfamilies. JMJD2 uses methylated H3K9 and H3K36 as demethylating substrates. Besides histone, JMJD2B takes part in the regulation of AKT1 activity by demethylation [5,6,7]. 

The typical members of the JMJD protein family contain a signature HX(D/E)XnH sequence motif capable of complexing Fe^2+^. The cofactors 2-OG, Fe^2+^, and oxygen are required for JMJD proteins to demethylate lysine or arginine residues. Succinate and carbon dioxide are examples of byproducts. In addition to their demethylase activity, JMJD proteins have also been shown to have hydroxylase activity toward RNA as well as to asparagine, aspartate, histidine, lysine, and arginine residues [4,8,9,10,11,12,13,14]. For the catalysis details of KDMs, some computational studies about KDM4A and PHF8 (JMJD7B) provide valuable information [15,16,17]. Using combined quantum mechanics/molecular mechanics (QM/MM) and molecular dynamics (MD) simulations, the catalytic features of PHF8, including dioxygen activation, 2OG binding modes, and substrate demethylation processes, were investigated. Both iron center rearrangement and conformational modulation of substrate orientation play crucial roles in the PHF8 catalysis process. Results for KDM4A demonstrate that molecular orbital control, correlated movements of the protein and histone substrates, and the substrate binding mechanism all work in concert to increase the reactivity of the Fe (IV)-oxo intermediate. Additionally, using analysis of dynamics cross-correlation analysis (DCCA), the possible impact in the second coordination sphere (SCS) and long-range (LR) areas on the hydrogen atom transfer (HAT) step of PHF8 were investigated in addition to the enzyme-specific first-sphere catalytic residues. The findings illustrate the ability of the SCS and LR residues to modulate the molecular orbital mechanism, the activation barrier for HAT, and product stability.

Some JMJD proteins with molecular weights greater than 100 kDa have both the JmjC and some additional conserved domains including JmjN and plant homeodomain (PHD), but some JMJC proteins with lesser molecular weights only have the JmjC domain [9]. More than the demethylase and hydroxylase capabilities outlined above, these JmjC domain-only proteins have a variety of functions. JMJD proteins, such as JMJD5, JMJD6 and JMJD7, have also been found to exhibit proteolytic clipping and tyrosine phosphorylation activity [18,19,20].

JMJD8 is a member of the JmjC domain-only group. Until recently, there has been no information about JMJD8’s enzymatic activity. Some researchers predict that JMJD8 may not possess any because the JmjC domain of JMJD8 displays an HXHXnH motif instead of HX(D/E)XnH [21]. JMJD8’s functions in cells and mice have been detailed in a few articles. JMJD8 acts as a positive regulator of the TNF-induced NF-κB signaling pathway [22]. By interacting with pyruvate kinase M2, JMJD8 plays a role in angiogenesis and cellular metabolism [23]. Downregulation of JMJD8 in DU145 prostate cancer cells resulted in decreased viability [24]. Similarly, in HEK293T cells, JMJD8 downregulation exacerbates tumor necrosis factor-induced apoptosis [22]. Additionally, JMJD8 knockdown has been demonstrated to significantly reduce the invasion of squamous cell carcinoma cells [25]. Recent studies have shown that JMJD8 is a novel molecular nexus between adipocyte intrinsic inflammation and insulin resistance. A JMJD8 gain of function was sufficient enough to drive insulin resistance, whereas loss of function improved insulin sensitivity in mouse and human adipocytes [26]. 

Despite these findings, it has been shown that reduced JMJD8 expression increases activation of the AKT/NF-B/COX-2 signaling pathway, which promotes cell proliferation and the repair of DSBs in cancer cells [27]. Furthermore, it was discovered that knocking down JMJD8 increased the interaction of SETDB1 and phosphoinositide-dependent kinase 1 (PDK1) with AKT1, resulting in increased trimethylation of AKT1 at lysine 142 (K142), which is crucial for cell membrane recruitment, phosphorylation, and activation of AKT [28]. This implies that JMJD8 regulates tumor migration and invasion via altering AKT methylation and activation.

The main point of contention is whether JMJD8 is a pro- or anti-oncoprotein. These differences could be caused by various cell types, external stimuli, or a complicated control of other signaling molecules. In contrast, one of the crucial elements for JMJD8’s functionality is thought to be the regulation of AKT1. To explore the hypothesis that JMJD8 acts as a 2-oxoglutarate-dependent demethylase in epigenetically modulating the activation of AKT1, we expressed the JMJD8 protein and tested the enzyme’s activity toward tri-methyllysine of AKT1. We reported the demethylase activity of JMJD8 toward methylated AKT1 peptide and full-length protein and explored its relative functions in cells.

## 2. Results

### 2.1. JMJD8 Has KDM Activity

To test whether the JmjC domain-only protein JMJD8 can act as a KDM, we produced JMJD8 in *E. coli.* The JMJD8 protein was purified by nickel affinity column and gel filtration (Superdex 200) (Figure S1). The purified protein was frozen in −80 refrigerator for further use.

Firstly, we tested the demethylase activity of JMJM8 using the JMJD2 demethylase activity assay kit (Epigentek, P-3081). The fluorescence of the JMJD8 sample well was 5.8 × 10^7^ ± 0.6, which is almost twice that of the standard well with two nanograms of demethylated substrate (2.3 × 10^7^ ± 0.4). This finding demonstrated that JMJD8 can demethylase the tri-methylated lysine histone substrates provided in the kit.

JMJD8 has been identified as an endoplasmic reticulum protein that regulates AKT1 activity in various cell lines. The degree of methylation of various lysines influences AKT1 activity [5,6,7]. We chose one of the most important trimethylated lysines, K142, for further study. A synthetic segment of AKT1 with a trimethylated lysine residue (named AKT1-K142me3, corresponding to K142me3) was used to investigate the demethylation activity in vitro. To test demethylation, the reactions were examined using LC–mass spectrometry.

**Figure 1:**
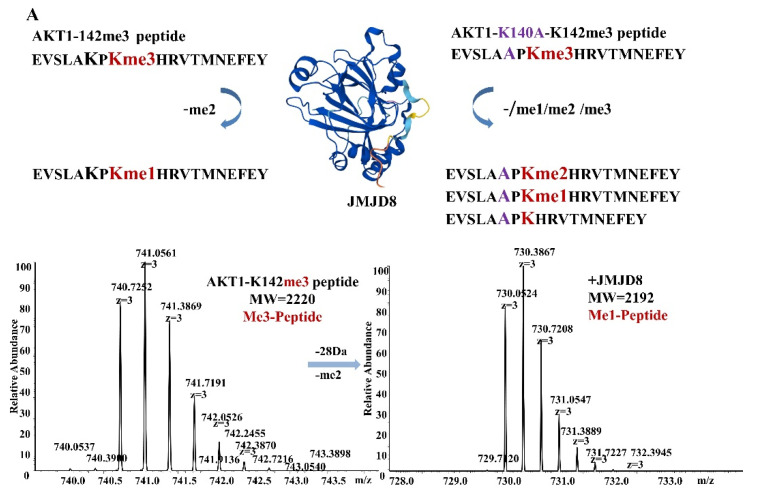

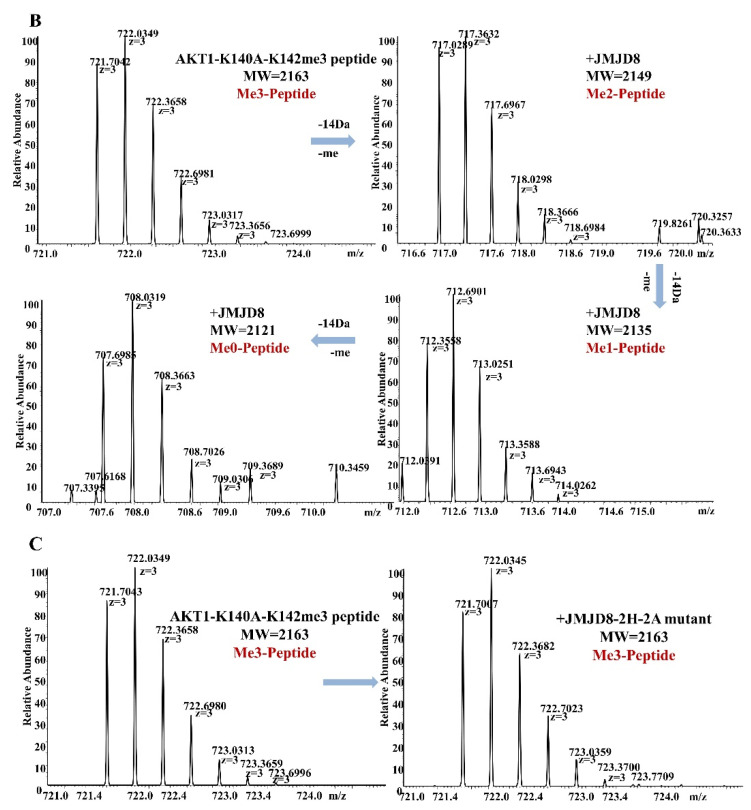
LC–MS analysis of demethylation of trimethylated lysine AKT1 peptides (AKT1-K142me3 (**A**) and AKT1-K140A-K142me3 (**B**)) by truncated recombinant catalytic domain constructs of JMJD8. (**C**) LC-MS analysis of demethylation of trimethylated lysine AKT1 peptides (AKT1-K140A-K142me3) by JMJD8-2H-2A mutant.

After 1 h incubation of JMJD8 with its appropriate substrate and co-factors, demethylation was found for both trimethylated peptides of AKT1 and its mutant. Product peptide spectra for demethylation of wild-type AKT1 peptide showed a mass shift of −28 Da, which corresponds to the elimination of two methyl groups (Figure 1A). In contrast, for the K to A mutant AKT1 peptide (corresponding to AKT1-K140A-K142me3), product peptide spectra manifested a mass shift of 14 to −42 Da that corresponds to the loss of one methyl group to three methyl groups (Figure 1B). The cofactor- or enzyme-free control samples did not exhibit any mass changes. Suyao and Yeo’s findings suggest that histidines (H179 H181 and H248) located in the center of the catalytic active site may be crucial in regulating the activity of JMJD8. The demethylase activity of JMJD8 was essentially eliminated by the mutation of H179 and H181 to alanine (JMJD8-2H-2A) (Figure 1C).

### 2.2. NMR Analysis of KDM Activity

**Figure 2:**
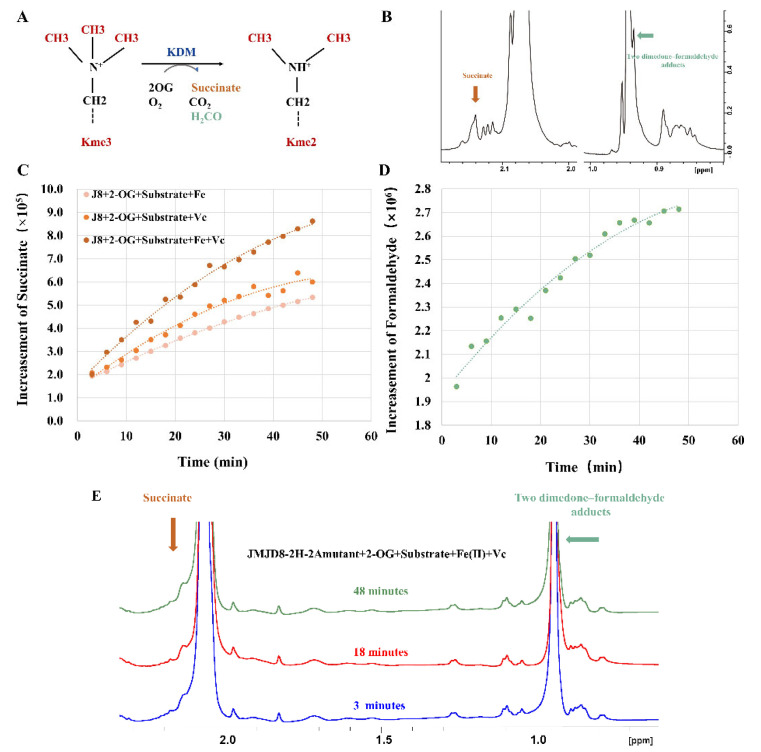
NMR analysis of demethylase activity. (**A**) Oxygen- and 2OG-dependent catalytic activities displayed by JMJD proteins toward tri-methylated lysine residue with coproduct succinate, carbon dioxide and formaldehyde. (**B**) ^1^H NMR spectrum of the JMJD8-catalysed demethylation of AKT-K140A-K142me3. (**C**) Graphs showing the degree of succinate production catalyzed by JMJD8 as quantified by ^1^H NMR (600 MHz). (**D**) Detection of an increment of formaldehyde release during JMJD8-catalysed trimethylated lysine demethylation. Dimedone reacts with formaldehyde in aqueous solution to form stable adducts that are detectable using ^1^H NMR. Incubation of a reaction mixture containing JMJD8 (6.7 μM), AKT1-K142me3 peptide (250 μM), 2OG (0.2 mM), ascorbate (0.5 mM), iron (II) (0.1 mM) and dimedone (1 mM) revealed the formation of two dimedone adducts using ^1^H NMR (600 MHz). (**E**) Time-dependent ^1^H NMR spectrum showing that no detectable succinate or formaldehyde was generated during the demethylation of AKT-K140A-K142me3 mediated by the JMJD8-2H-2A mutant.

We employed NMR spectroscopy to follow JMJD8-catalyzed demethylation of the AKT1-K142Me3 peptides to confirm the MS results. Immediately after initiation of the reaction, the samples containing JMJD8, with trimethylated lysines AKT1-K142me3 peptide substrate and cofactors/co-substrates, were transferred to NMR tubes and then subjected to ^1^H NMR (600 MHz) analyses. Production of succinate was evidenced by an increase in a 1 H resonance at 1 H 2.15 p.p.m. The degree of succinate generation was higher in the presence of the peptide and all three cofactors than in the absence of ascorbate or Fe^2+^, implying that all three cofactors play a key role in the reaction (Figure 2A).

The addition of the formaldehyde-trapping reagent dimedone to the NMR reaction mixture in the presence of the AKT1-K142me3 peptide resulted in the emergence of dimedone–formaldehyde adducts. These adducts have been previously observed to form upon incubation of dimedone with formaldehyde released during JmjC KDM catalyzed demethylation (that is, methyl group oxidation to release formaldehyde) [29]. The enzymatic reaction process was also recorded by the dimedone–formaldehyde adducts (Figure 2B). The Km of JMJD8 for the AKT1-K142me3 peptide was about 17.4 μM, and the Km value of JMJD8 was lower than that of KDM4B reported. The Km of KDM4B/JMJD2B was about 88.3 or 31 μM using an H3K9me3 peptide as the substrate [30,31]. The combined MS and NMR results thus reveal that JMJD8 can catalyze tri-methyl lysine demethylation.

### 2.3. Demethylation of Methylated Full-Length AKT1

**Figure 3:**
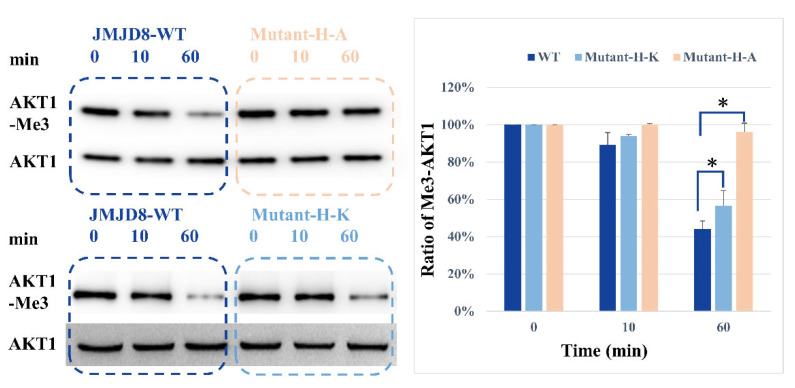
Western blotting analysis of demethylase activity toward full-length AKT1. Western blotting analysis was used to estimate the extent of demethylation of the full-length AKT1 protein by JMJD8 and its mutant (Mutant-2H-2K, Mutant-2H-2A) in vitro. The remaining trimethylated lysine was labeled by a specific antibody after the reaction of demethylation. The reaction was carried out for 0, 10 and 60 min with JMJD8, methylated AKT1 and cofactors mixing together. The right panel shows the relative ratio of remaining trimethylated-AKT1 protein analyzed by ImageJ software. Data were pooled from three independent experiments, and the results are represented as mean ± SD. * *p* < 0.05.

Given the ability of JMJD8 to catalyze demethylation of lysine residues in synthetic peptide sequences, we are also interested in investigating whether it could catalyze lysine residues in “natural” positions in AKT1. AKT1 protein was expressed using 293F cells by transforming vectors pCDNA 3.1-HA-AKT, and EGF (10 ng/mL) was added to the medium to induce the methylation of AKT1. The HA-AKT1 protein is purified by the HA-affinity column. Here, the WB results show that JMJD8 can lower AKT1’s tri-methyl lysine level. We also tested the demethylase activities of histidine-mutant (H179K, H181K) JMJD8 and alanine-mutant (H179A, H181A) against tri-methylated AKT1. Compared with wide-type JMJD8, time-dependent results indicate that the JMJD8-2H-2K mutant has lower demethylase activity (Figure 3) and the JMJD8-2H-2A mutant has little demethylase activity. The JMJD8-2H-2K mutation from histidine to lysine with the change of the positive charged side chain may alter the binding of co-factors and the efficacy of demethylation. Taken together, these data suggest that JMJD8 could demethylate the tri-methyl lysine of AKT1.

### 2.4. JMJD8 Modulates the Activity of AKT1 in H1299 Cells

**Figure 4:**
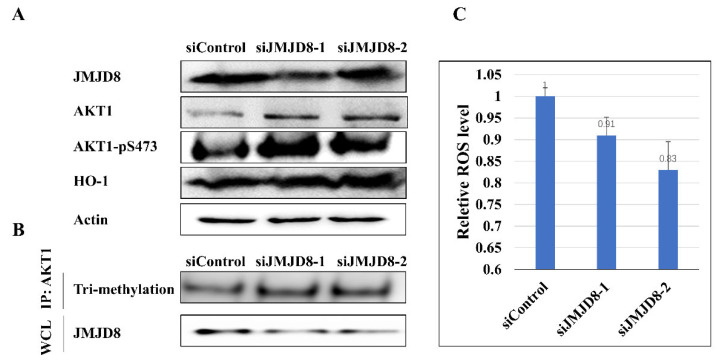
JMJD8 regulated the activity of AKT1 in H1299 cells. (**A**) Levels of AKT1, p-AKT(Ser473) and HO-1 were detected in JMJD8-knockdown cells. (**B**) Level of tri-methylation of AKT1 in JMJD8-knockdown cells. Cells were transfected with siJMJD8, and AKT1 plasmids and whole cell lysate (WCL) were collected for IP with AKT antibody, followed by IB analysis. (**C**) Ros level in H1299 cells was assessed. Knockdown of JMJD8 helped the remove ROS in cells.

Lysine methylation of non-histone proteins is involved in numerous molecular events, including protein–protein interaction, protein stability, protein subcellular localization, and transcription [32]. The trimethylation of AKT aids in the enhancement of AKT membrane localization and phosphorylation [33]. Given the activity of demethylase of JMJD8 toward AKT1, it can be inferred that JMJD8 may have an impact on AKT1’s function in cells. We next set out to determine whether the amount of JMJD8 can fine-tune the activity of AKT1. The results showed that siRNA-mediated reduction of JMJD8 expression increased AKT1 expression and improved AKT1 phosphorylation (Ser 473). The results of cells transfected with siJMJD8 and AKT1 plasmids showed that JMJD8 knockdown promoted the tri-methylation of AKT1 (Figure 4B and Figure S2). In addition, enhanced AKT1 activity resulted in a rise in heme oxygenase-1 (HO-1) in the downstream signal molecular (Figure 4A). Finally, the total amount of ROS (reactive oxygen species) in the cells reduced.

It has been reported that AKT1 methylation in its linker region is mediated by the histone methyltransferase SETDB1, which is antagonized by the demethylase KDM4B (JMJD2B) [5]. Likely, Suyao’s results showed that JMJD8 knockdown promoted the trimethylation of AKT1, while JMJD8 overexpression inhibited this modification [28]. All the results indicate that AKT1 activity is regulated in a methylation-dependent way. Both KDM4B and JMJD8 may take part in the regulation of AKT1 methylation level.

## 3. Discussion

**Figure 5:**
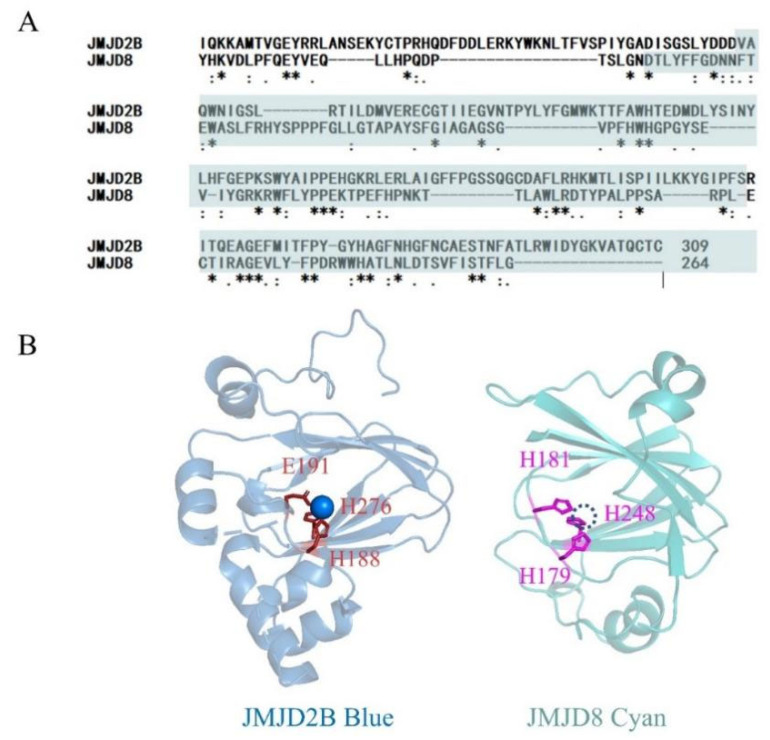
Sequence alignment and structural superimposition of JmjC domain of JMJD8 with JMJD2B. (**A**) Sequence alignment of JMJD2B and JMJD8 by Clustal Omega. JmjC domain is highlighted in cyan. An * (asterisk) indicates positions which have a single, fully conserved residue. ** indicates positions which have two fully conserved residues. *** indicates positions which have three fully conserved residues. A : (colon) indicates conservation between groups of strongly similar properties. A . (period) indicates conservation between groups of weakly similar properties. (**B**) Structural superimposition of JmjC domain of JMJD8 (AlphaFold: AF-Q96S16-F1) with JMJD2B(PDB:7JM5). The JMJD8 is shown as cyan carbon atoms. The JMJD2B is shown as blue carbon atoms with a blue Ni(II). Ni(II) is used to substitute Fe(II) in the crystal of JMJD2B.

JMJD8 belongs to a subfamily of tiny JMJD proteins that are evolutionarily distant and have molecular weights ranging from 27 to 71 kDa. The functions of these relatively small proteins include hydroxylation, histone demethylation, endo peptidase at lysine/arginine-methylated, etc. JMJD8, found several years ago, is mentioned in several papers, but the enzymatic activity of JMJD8 is rarely reported. JMJD8 studies in carcinoma cells show conflicting results. Some findings suggest that JMJD8 plays a pro-cancer role, while others suggest that it plays an anti-cancer role [22,23,24,25,26,27]. We postulated that JMJD8 may operate as a demethylase to affect the activity of other proteins based on the presence of the JmjC domain in the sequence and research showing that aberrant JMJD8 expression enhances AKT activation. An alignment of predicted structure of JMJD8 with the structure of JMJD2 reveals homology between the two JmjC proteins (Figure 5). In the enzyme catalyzed core, the Fe (II)-coordinating His-X-His-Xn-His triad of JMJD8 is similar to the His-X-Asp-Xn-His triad of JMJD2. The structural similarity indicates the structural basis of JMJD8 with lysine demethylase activity. Consistently, the JMJD8-2H-2A mutation almost lost the demethylase activity similarly to JMJD2 [34].

Proteins are regulated by an incredible array of post-translational modifications (PTMs). The methylation of lysine residues on histone proteins is a PTM that has long been known to play a role in chromatin and epigenetic events. Aside from histone, lysine methylation has been detected in hundreds, if not thousands, of non-histone proteins. Lysine methylation regulates major cellular mechanisms involved in cancer, such as growth signaling and the DNA damage response [32]. The degree of lysine methylation influences the activity of AKT1, a critical protein in the PI3K-AKT pathway [7]. The enzymes that catalyze lysine methylation, the enzymes that remove lysine methylation, and the cancer pathways known to be regulated by methylation are the main issues.

We focus on the demethylation of AKT1-K142me3 by JMJD8. First, we defined the enzymatic activity of JMJD8 toward tri-methylated peptide. There are discrepancies between wild type AKT1 peptides and mutant AKT1 peptides in the demethylated products. The adjacent K to A mutation could cause variations in the binding orientation between the substrate and enzyme, finally bringing about distinct products.

We then estimated the demethylation of tri-methylated lysine in the whole protein. The findings support the hypothesis that JMJD8 is a trimethylated lysine-based demethylase. According to Guo et al., AKT is activated by SETDB1-mediated lysine methylation, which is inhibited by the Jumonji-family demethylase KDM4B [5]. Our findings show that inhibiting JMJD8, a demethylase that eliminates AKT tri-methylation, increases AKT1 activity. Despite the consistency of the results, it is still unknown how JMJD8 and KDM4B are involved in demethylation. Different demethylated proteins may control the timing and level of lysine methylation at various locations on the AKT1 protein.

JMJD8 may also have other demethylase activities, as well as hydroxylation and protease activities. This needs the screening of catalytic substrates of JMJD8 or the biomolecules that interact with JMJD8 to confirm the enzymatic activity in vitro and in vivo. Further research also includes investigating the biological processes and cancer mechanisms potentially regulated by the multitude of lysine methylation sites. The demethylation of trimethylated lysine by JMJD8 is the subject of this study. Numerous proteins have been shown to include dimethylated or monomethylated lysine and methylated arginine. More research is needed to fully understand JMJD8’s enzymatic activity in those PTMs.

## 4. Materials and Methods

### 4.1. Cell Culture

H1299 cells were purchased from the American Type Culture Collection (Manassas, VA, USA). H1299 cells were maintained in Dulbecco’s modified Eagle’s medium (DMEM; Gibco, Thermo Fisher Scientific, Inc., Waltham, MA, USA) supplemented with 10% (*v*/*v*) fetal bovine serum (FBS), 100 IU/mL penicillin and 100 µg/mL streptomycin. Cells were grown in a humidified 37 °C incubator with 5% CO_2_.

Expi293 cells were acquired from Union-Biotech (Shanghai, China) and maintained in shaking incubators at 37 °C, humid atmosphere and 8% CO_2_ in a serum-free proprietary culture medium (Union-293 UP1000, chemically defined medium with L-glutamate).

### 4.2. Plasmid and Expression Vectors

The JMJD8(27-264aa) expression construct was produced by inserting the coding sequences of JMJD8 into the pET28a plasmid. The N-terminal HA-tagged AKT1 overexpression construct was produced by inserting the coding sequences of AKT1 into the pcDNA3.1 plasmid. JMJD8 mutant was generated by PCR. All constructs used in this study were verified by sequencing.

JMJD8 was produced as an N-terminally His6-tagged protein in Escherichia coli cells. Proteins were purified by Ni-affinity chromatography and size exclusion chromatography. Full-length HA-tagged AKT1 was expressed in Expi293 cells by transiently transfect plasmids encoding full-length AKT1(1–480) using polyethylenimine solution (PEI) (Polysciences, Warrington, PA, USA) as transfect agent [35].

### 4.3. Reagents and Antibodies

Trimethylated peptides were purchased from BankPeptide Inc. (Hefei, China). Antibodies against AKT1 (cat. no. 2938), pAKT (cat. no. Ser473) (4060), tri-methyl lysine motif (cat. no. 14680) and -actin (4970) were purchased from Cell Signaling Technology (Danvers, MA, USA). Antibody against JMJD8 (sc-515520) and HO1(10701-1-AP) was purchased from Santa Cruz Biotechnology ((Dallas, TX, USA) and Proteintech (Rosemont, IL, USA)). Horseradish peroxidase-conjugated secondary antibodies were purchased from Cell Signaling Technology. Recombinant human EGF was purchased from PeproTech (Rocky Hill, NJ, USA). The sequences of JMJD8 siRNA oligos and negative control siRNA are listed in Table 1.

### 4.4. SiRNA Transfection

Transfections of siRNAs were performed using X-tremeGENE HP DNA Transfection Reagent (Roche, Manheim, Germany) following the manufacturer’s protocol. Corresponding cells carrying scramble siRNA plasmids were used as control.

### 4.5. Activity Assays

#### 4.5.1. LC-MS Activity Assays

Recombinant proteins were incubated with 10 mM peptides (and cofactors/substrates) for 1 h at 37 °C, unless otherwise specified. All assays were performed in 20 mM Tris (pH 7.5) buffer with cofactors 2OG (200 μM), ascorbate (500 μM) and iron (II) (100 μM). Reactions were quenched by placing the mixture in liquid nitrogen immediately and analyzed with LC-MS. Negative controls without enzyme were included. Note that in some cases, small variations (≤2 Da) in the absolute experimentally observed values compared with the calculated values for peptides were observed. However, in most cases where demethylation is assigned, the mass shift was 14 Da.

#### 4.5.2. NMR Assays

NMR spectroscopy was carried out as described [14]. All the NMR spectra were recorded at 298 K using a Bruker Avance AVIII 600 MHz spectrometer equipped with cryogenic probes, optimized for 1 H observation and installed with the Topspin software.

The JMJD8 used in NMR assay is about 10 μM, and the substrate was 100 μM. All samples were prepared in Eppendorf tubes (500 μL volume) before being transferred to 5 mm NMR tubes (Norell, Morganton, NC, USA); time course data were then collected over a period of 60 min at 3 min intervals using an automated routine. Enzyme stocks were diluted with deuterated Tris (Tris-d11, Cambridge Isotope Laboratories, Tewksbury, MA, USA) buffer (prepared by diluting 500 mM tris-d11, 5 M NaCl in D2O pH 7.5) when added to the samples. Chemical shifts are reported relative to the solvent water resonance.

For peptide Km determination, NMR-based assays were carried out over a range AKT1-K140A-K142me3 (5–200 µM) concentrations. The initial slope for enzyme progress curves was taken for each peptide concentration, and data were fitted to the Michaelis–Menten equation in GraphPad Prism 8 (GraphPad Software, San Diego, CA, USA).

#### 4.5.3. WB Assay

Recombinant JMJD8 proteins were incubated with purified tri-methylated AKT1 and cofactors/substrates for different times at 37 °C. Reactions were quenched with 6* protein loading buffer and analyzed with Western blotting. Negative controls included were samples incubated for 0 min.

### 4.6. Western Blotting Analysis and Immunoprecipitation

Cells were washed with cold PBS, and total cell lysate was prepared with radio-immunoprecipitation assay (RIPA) buffer containing protease inhibitors and protein phosphatase inhibitors. Protein concentrations were determined using a Pierce™ BCA Protein Assay Kit (Thermo Scientific™, Waltham, MA, USA). Then, 50 μg cell lysate was resolved using 10% or 12% SDS-PAGE and transferred onto a polyvinylidene fluoride (PVDF) membrane. Following blocking in Tris-buffered saline with 0.1% Tween 20 (TBS-T) and 1% skim milk, the PVDF membrane was incubated with primary antibody at 4 °C overnight with gentle agitation. Subsequently, the membrane was washed three times with TBS-T and incubated with corresponding horseradish peroxidase-conjugated secondary antibodies for 2 h at room temperature. The protein bands were visualized using enhanced chemiluminescent substrate (Pierce™ ECL Western Blotting Substrate, Thermo Scientific™, Waltham, MA, USA) and visualized. Relative protein expression levels were analyzed using ImageJ software.

For immunoprecipitation analysis, 1000 μg lysates were incubated with the AKT1 antibody (1–2 μg) for 2–4 h at 4 °C followed by 1 h incubation with Protein A/G sepharose dynabeads (Invitrogen, Carlsbad, CA, USA). The recovered immunocomplexes were washed five times with NETN buffer (20 mM Tris (pH 8.0), 150 mM NaCl, 1 mM EDTA and 0.5% NP-40) before being resolved by SDS–PAGE and immunoblotted with indicated antibodies. Quantification of immunoblot band intensities was performed using Image J software.

## Figures and Tables

**Table 1:** siRNA sequences.

siRNA	Sequences (5′-3′)
Negative control siRNA	UUCUCCGAACGUGUCACGU
si-JMJD8-1	GACUUGCCCUUCCAGGAGU
si-JMJD8-2	GUCGUAAGCGCUGGUUCCU

## Data Availability

Not applicable.

## Supplementary Materials

The following supporting information can be downloaded at: https://www.mdpi.com/article/10.3390/ijms24010460/s1.
