# Supplementary material for: JMJD8 Functions as a Novel AKT1 Lysine Demethylase
Source: Int J Mol Sci. 2022 Dec 27;24(1):460. doi: 10.3390/ijms24010460 (PMC9820096; doi:10.3390/ijms24010460)

Supplementary Information

Supplementary Figure 1. SDS-PAGE of purified JMJD8 protein

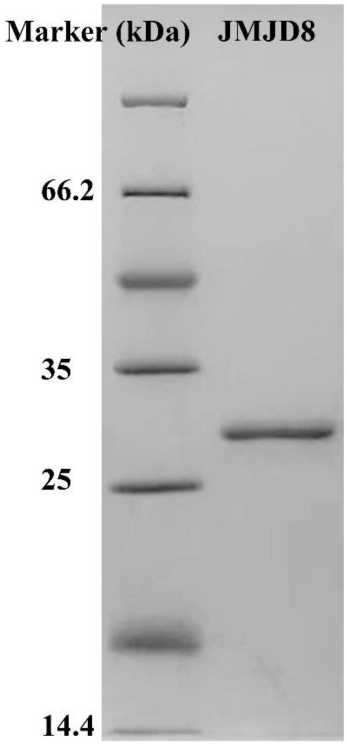

Supplementary Figure 2. Immunoprecipitation of AKT1.

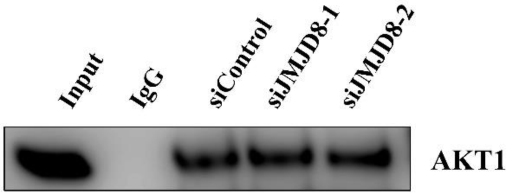

Supplement: Supplementary file 1 [file ijms-24-00460-s001.zip › ijms-2005222-supplementary.pdf]
